# Atomic force microscopy-based photothermal infrared microscopy for aqueous environments using graphene-based microfluidic cells

**DOI:** 10.1039/d5na01148e

**Published:** 2026-01-26

**Authors:** Yasuhiko Fujita, Mariko Takahashi, Hirohmi Watanabe

**Affiliations:** a Research Institute for Sustainable Chemistry, National Institute of Advanced Industrial Science and Technology (AIST) Kagamiyama 3-11-32 Higashihiroshima 739-0046 Japan Yasuhiko.fujita@aist.go.jp

## Abstract

We present the first demonstration of atomic force microscopy-based photothermal-induced resonance (PTIR) measurements of hydrated polymers under aqueous conditions, utilizing microfluidic cells with a graphene layer as an atomically thin IR-transparent window. Our findings show that polymer swelling can be successfully detected through changes in the PTIR spectrum.

## Abstract 2

This study presents the first implementation of AFM-based photothermal IR microscopy, integrated with a graphene-based microfluidic system for polymer analysis in aqueous environments.

## Graphical abstract

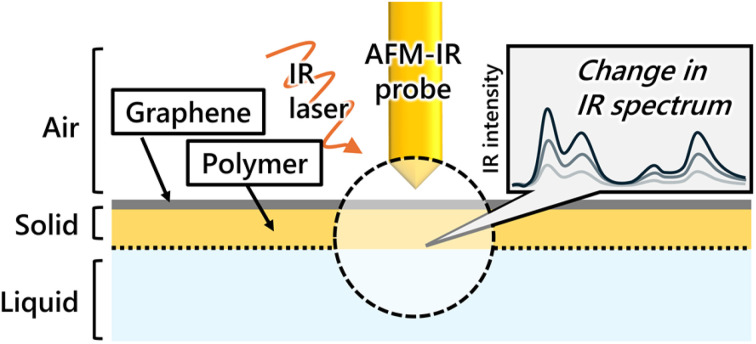

Polymers exhibit active chain motion even at room temperature, and their mobility is significantly influenced by the surrounding environment. As social demands continue to increase, polymers designed for aqueous environments—such as biodegradable^1–3^ and biomedical^4^ polymers—are becoming increasingly important. Therefore, conducting structural analysis in relevant environmental settings is essential.

Several analytical techniques, including X-ray photoelectron spectroscopy,^5^ secondary ion mass spectrometry,^6,7^ and transmission electron microscopy^8,9^ (TEM), along with the recent advancement of nanoscale infrared (IR) microscopy,^10,11^ have facilitated detailed characterization of polymer chemical structures at the nanoscale. However, these techniques often face limitations when applied under aqueous conditions, presenting a significant challenge for effective structural analysis in appropriate environments.

To address these challenges, researchers have explored various analytical techniques for aqueous environments. One notable method is atomic force microscopy (AFM). For instance, advanced AFM techniques, such as high-speed AFM and AFM-force curve spectroscopy, have demonstrated dynamic visualization of active motion of biomolecules^12–15^ or analysis of DNA interactions^16,17^ in liquid environments. While these methodologies can provide insights into the nanoscale morphological and mechanical properties of targeted samples under aqueous conditions, they do not provide detailed chemical information essential for a mechanistic study.

To overcome this limitation, innovative methods that integrate scanning probe microscopy with optical spectroscopy have been developed. One such method is the nano-Fourier-transform infrared (FTIR) technique,^18^ which combines FTIR spectroscopy with atomic force microscopy (AFM) by using metallic probes acting as mid-IR antennae. In liquid AFM configurations where an aqueous solution is applied directly to the tip-sample system, nano-FTIR and related techniques have proven effective for nanoscale IR imaging of various systems in aqueous environments, including polaritons,^19,20^ polymers,^21,22^ and biomolecules.^22–25^ Furthermore, recent advancements have also explored the use of ultra-thin IR windows, such as graphene,^26–29^ or silicon nitride^23^ membranes, to address the decrease in the quality factor (*Q*-factor) that typically occurs in liquid AFM measurements.^21^ While these alternative methodologies hold promise for analyzing various target materials, they also present significant challenges in terms of sensitivity. Specifically, nano-FTIR detects IR signals not only from the target molecules but also from the surrounding solvent, leading to increased background interference due to water's large IR absorption, which ultimately reduces effective sensitivity (Fig. 1).

**Fig. 1:**
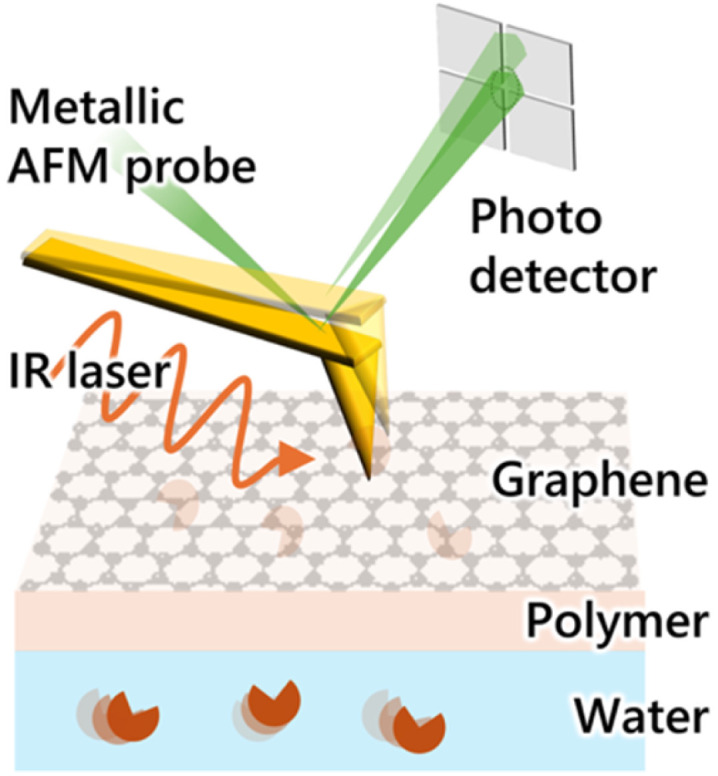
Conceptual illustration of PTIR microscopy for aqueous environments using graphene-based microfluidic cells.

Among various IR techniques, mid-IR photothermal (MIP) spectroscopy shows particular promise in addressing the issue of solvent IR absorption. In optical-MIP spectroscopy, pulsed IR irradiation induces a thermal lens effect, and changes in the reflectance of the target sample are detected *via* a focused visible laser beam. Notably, due to the rapid heat diffusion properties in liquids, the IR signal emanating from solid samples is substantially stronger than that from the surrounding solvent.^30^ This principle has underpinned recent demonstrations of sub-µm resolution IR imaging of living cells in aqueous environments, marking a breakthrough in label-free, non-destructive measurements of biological samples.^31–35^ Hence, integrating AFM-based MIP spectroscopy,^11^ commonly known as PTIR microscopy, with thin IR-transparent membranes could allow for high-sensitivity and high-resolution nanoscale IR measurements of polymers in liquid environments. This integration would effectively suppress the strong signals typically generated by solvents. Although this advantage is expected, no experimental demonstrations of this integrated approach have been reported so far.

This study presents the first demonstration of PTIR measurements of polymers under aqueous conditions utilizing an atomically thin, IR-transparent membrane made of graphene. Our methodology involves coating the target polymer on one side of the graphene, while photothermal IR signals of the polymer are collected from the opposite side through the graphene. This approach allows for the detection of IR signals from polymers under aqueous conditions without experiencing the decrease in the signal-to-noise ratio caused by cantilever damping due to the immersion of the cantilever in water and by significant background IR signals from water, allowing for analysis of heterogeneity in the swelling behavior of polymers with sub-100 nm resolution. The main innovation presented in this study is a methodology developed for PTIR measurements that is uniquely integrated with a graphene window. Specifically, a gold-coated AFM probe is engaged to the graphene-exposed side, where a pulsed IR laser, modulated at the 7th cantilever resonance frequency (1.5–2 MHz for the cantilever used), was irradiated onto the tip-sample system. The subsequent deflection changes of the cantilever, which correlate with the polymer's photothermal IR signal that has passed through the graphene layer, were monitored using a lock-in amplifier (*i.e.*, homodyne AFM-IR). With our measurement conditions, depth resolution is expected to be approximately 100 nm.^36^

For this purpose, we developed a microfluidic flow cell using a commercial graphene TEM grid, which we refer to as graphene cells (see Fig. S1). The fabrication process for the graphene cells included laser cutting of acrylic plates and heat-sealing films, followed by assembling these components together with the graphene TEM grid. The commercial graphene TEM grid utilized in this study contains a SiO_2_ membrane with 3 µm diameter pores, entirely covered with 6–8 layers of graphene (Fig. S2). This SiO_2_ and graphene membrane was supported by silicon nitride (SiN).

Initially, we verified the feasibility of detecting PTIR signals from polymers that exist underneath the graphene. We accomplished this by measuring PTIR spectra of a UV-curable acrylic polymer coated on graphene. Fig. 2 displays the spectra obtained through graphene (shown in blue, with the vertical axis magnified ×3) compared to those measured by directly engaging the polymer (shown in black), both assessed under dry conditions. Although we observed a noticeable decrease in signal intensity when measured through graphene, distinct polymer-derived IR absorption peaks were still evident, confirming the feasibility of PTIR detection through graphene. The observed IR signal attenuation may be attributed to IR absorption by graphene.

**Fig. 2:**
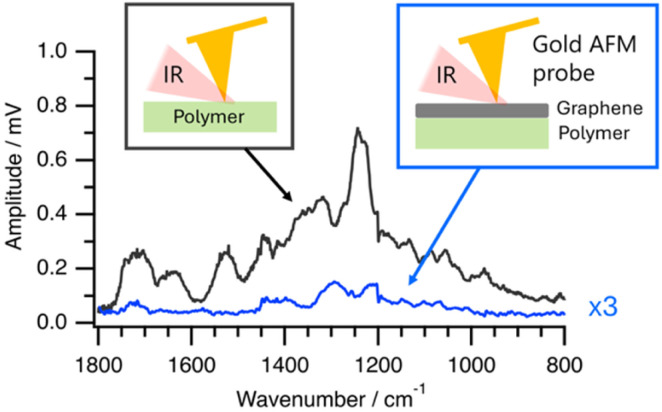
PTIR spectra of an acryl-based UV-resin polymer measured in direct contact with the polymer surface (black) and through the graphene layer (blue, ×3 magnified).

To assess the spatial resolving capability of PTIR signals through graphene, we immobilized polystyrene (PS) beads (average diameter: 300 nm) on the graphene surface and conducted PTIR spectral and imaging measurements, again under dry conditions. As illustrated in Fig. 3a, the PS beads were visible in the graphene-exposed region in the AFM height images, likely due to the formation of wrinkles in the graphene upon the adsorption of PS particles.^37^ The spectra collected from the PS beads shown in Fig. S3 demonstrated characteristic IR absorptions of PS (1492 cm^−1^ for C=C stretching and 1452 cm^−1^ for CH stretching), further confirming successful signal acquisition through graphene. IR images at 1492 cm^−1^, shown in Fig. 3b, obtained at a square area indicated in Fig. 3a, showed distinctly resolved individual PS beads, achieving a spatial resolution comparable to observations made without the graphene layer (Fig. S4). Note that control IR imaging at off-resonance (1550 cm^−1^) and without laser irradiation, as shown in Fig. S5, only showed nearly homogeneous signals due to graphene's IR absorption and/or dark noise from the system, suggesting that the distinct IR signals from PS were successfully measured. These findings highlight that the capability of high-resolution PTIR imaging is still represented even in the presence of a graphene window.

**Fig. 3:**
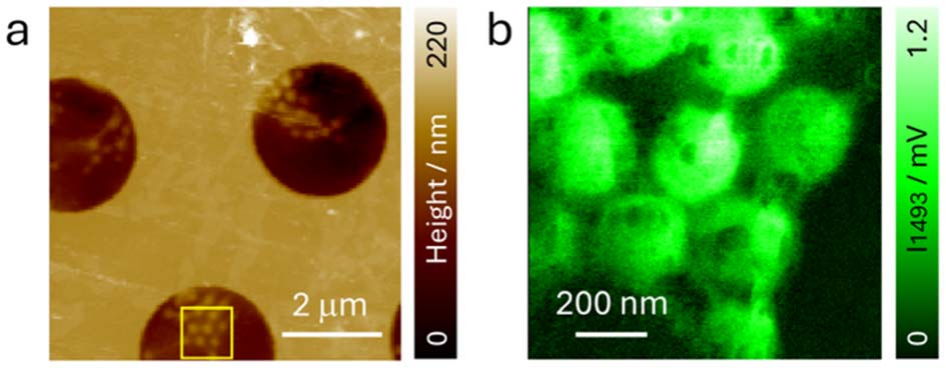
(a) AFM height image of a PS bead-immobilized graphene cell on the back side. (b) IR image at 1492 cm^−1^ obtained in the square region indicated in (a).

Lastly, we evaluated the performance of PTIR under aqueous conditions. A thin film of poly(2-hydroxyethyl methacrylate) (pHEMA), approximately 50 nm in thickness, was coated on the graphene TEM grids, and PTIR measurements were conducted in a water flow environment at a flow rate of 0.1 mL min^−1^ (Fig. S6). As the thickness of the pHEMA layer is below the PTIR depth resolution, PTIR detects IR signals from its entire thickness. The AFM-IR measurements were performed by obtaining IR images at 1724 cm^−1^ (C=O stretching of pHEMA) and 1705 cm^−1^ (hydrogen-bonded C=O stretching). These measurements were performed under dry conditions and during water flow at a rate of 0.1 mL min^−1^. Intensity ratio images (*I*_1724_/*I*_1705_) were generated and subsequently compared through analysis of the difference ratio images and corresponding histograms. The measurements were performed on the region depicted in the AFM image presented in Fig. 4a. IR images (measured at on-resonance: 1724 and 1705 cm^−1^ and off-resonance: 1840 cm^−1^) along with the associated AFM height images under both dry and wet conditions, as shown in Fig. S7, demonstrated the robustness of PTIR measurements under both dry and flow conditions. The comparative spectra of pHEMA under dry and aqueous conditions are shown in Fig. 4b. The spectrum shown here is an average of spectra from 3 sets of experiments, with each experiment measuring 5 spectra from different areas. In the aqueous state, we observed an increase in the relative intensity of the 1705 cm^−1^ band (assignments: hydrogen-bonded C=O stretching) compared to the 1724 cm^−1^ band (assignments: non-hydrogen-bonded C=O stretching). This result indicates that the carbonyl environment of pHEMA was modified due to the swelling by water,^38^ with similar spectral changes observed in bulk FTIR measurements (Fig. S8). Fig. 4c and d illustrate intensity ratio images (*I*_1705_/*I*_1724_) obtained under dry and wet conditions, respectively. The experiments began under wet conditions, with PTIR images collected after 45 minutes of flow. Following this, the sample was placed in a dry chamber for over 12 hours (relative humidity <1% and temperature 25 °C), after which dry measurements were conducted. Corresponding to the spectral alterations, an increase in the *I*_1705_/*I*_1724_ ratio was observed under the wet condition across the majority of the analyzed regions. The average increase in the *I*_1705_/*I*_1724_ ratio under wet conditions is further corroborated by the histogram presented in Fig. 4e, which was derived from each individual map. Moreover, the difference IR image, created by subtracting the *I*_1705_/*I*_1724_ image under dried conditions from that under wet conditions, reveals that the swelling induced by water absorption in pHEMA occurs predominantly across the measured area. Nonetheless, certain regions exhibited a degree of heterogeneity in the *I*_1705_/*I*_1724_ ratio, with some areas retaining values comparable to those under dried conditions. This observed heterogeneity in swelling could be attributed to variations in coating thickness or the interactions between pHEMA and the graphene substrate. Collectively, these findings demonstrate that integrating a graphene cell with PTIR enables stable and high-sensitivity IR signal acquisition under water flow conditions, as evidenced by the detection of hydrogen-bonding variations in polymers without interference from solvent signals.

**Fig. 4:**
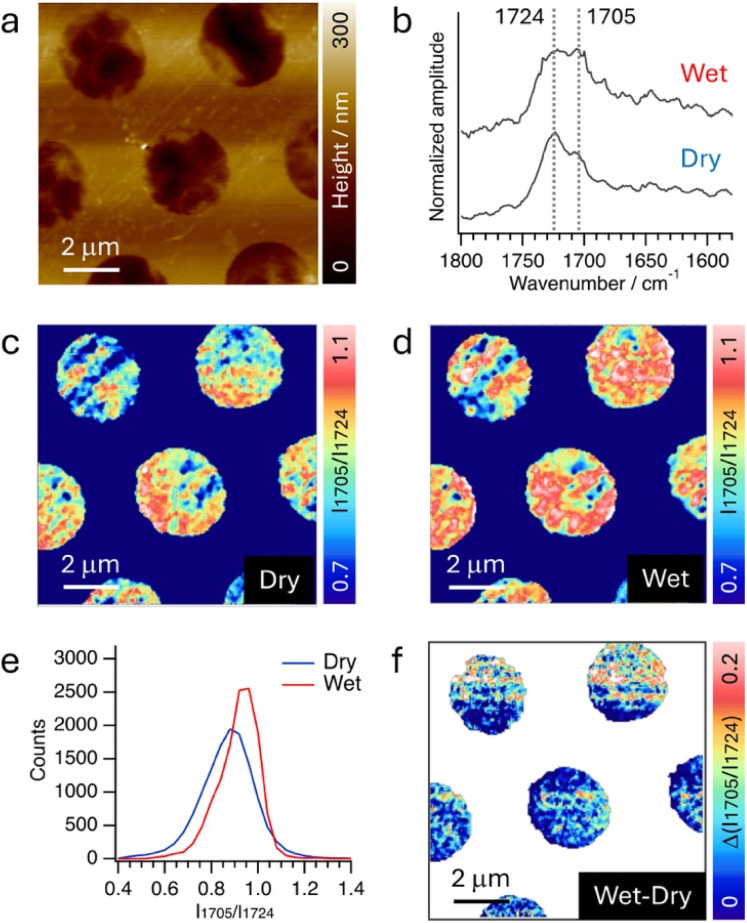
(a) AFM height image of the pHEMA-coated graphene cell on the back side obtained under water flow at 0.1 mL min^−1^ (b) PTIR spectra of pHEMA obtained under dry (bottom) and flow (top) conditions (c and d) Intensity ratio image (*I*_1705_/*I*_1724_) obtained under dried conditions (c) and during water flow (d). Both images were obtained in the same area. (e) Histogram of *I*_1705_/*I*_1724_ obtained under dry conditions (red) and during water flow (blue). (f) Difference intensity ratio image (wet – dry).

## Conclusion

In conclusion, we successfully developed a microfluidic flow cell using a graphene TEM grid, which facilitates PTIR measurements of polymers under aqueous conditions. Our findings indicate that, despite signal attenuation caused by graphene's IR absorption, we were still able to observe distinct IR signals from the polymers through the graphene. Furthermore, stable PTIR measurements can be conducted under flow conditions. This research underscores the effectiveness of PTIR microscopy for analyzing polymers in aqueous environments, opening new avenues for applications in materials science, nanotechnology, and biochemical sensing and enhancing our understanding of material behavior at the nanoscale.

## Author contributions

Y. F. designed the experiment. Y. F. and H. W. developed experimental resources. Y. F. and M. T. conducted MIP measurements. Y. F. supervised the whole project. All authors critically reviewed and revised the manuscript draft and approved the final version for submission.

## Conflicts of interest

There are no conflicts to declare.

## Supplementary Material

NA-008-D5NA01148E-s001

## Data Availability

All generated/analyzed data are available in this article or the supplementary information (SI). Additional data related to this paper are available from the corresponding author on reasonable request. Supplementary information is available. See DOI: https://doi.org/10.1039/d5na01148e.
