# Supplementary material for: Atomic force microscopy-based photothermal infrared microscopy for aqueous environments using graphene-based microfluidic cells
Source: Nanoscale Adv. 2026 Jan 26;8(5):1508–11. doi: 10.1039/d5na01148e (PMC12863398; doi:10.1039/d5na01148e)
Supplement: NA-008-D5NA01148E-s001 [file NA-008-D5NA01148E-s001.pdf]

Supplementary Information (SI) for Nanoscale Advances.
This journal is © The Royal Society of Chemistry 2026

Electronic Supplementary Information

Atomic force microscopy-based photothermal infrared microscopy for aqueous environments using graphene-based microfluidic cells

Yasuhiko Fujita *[1], Mariko Takahashi [1], Hirohmi Watanabe [1]

[1] Research institute for sustainable chemistry, National Institute of Advanced Industrial Science and Technology (AIST), Kagamiyama 3-11-32, Higashihiroshima 7390046 Japan.

Sample preparation

Fabrication of graphene cell: Acrylic plates (1mm thickness for bottom (Figure S1, E), 2mm thickness for top (Figure S1, C) and heat-sealing films (Parafilm® M, Bemis Flexible Packaging / Figure S1 A, D) were cut into the parts using a commercial CO_2 laser cutter (beamo 30W, FLUX Japan). Each component was assembled by heat-pressing using a Cu block on a hotplate set at 80 °C. After assembling the parts, tube connectors (Figure S1, B) were mounted in the holes and finally sealed with UV-resin. The maximum allowable flow rate of the fabricated graphene cell was typically 2 mL/min, as determined by testing with water for 30 minutes.

Polymer-coating to graphene: Before assembling the graphene cell, the target polymers were coated onto a graphene TEM grid (Figure S1a). Specifically, a polymer solution was drop-casted to the commercial graphene TEM grid (GN-6, EM Japan) under the following conditions:

Sample	Product	Company	Solvent	Concentration	Amount
UV resin	BD-SKEJ	BONDIC	-	-	10 uL
PS beads	Carboxyl Modified Latex Set (Diameter 300 nm)	Fujikura	Water	x100 dilution	10 uL
pHEMA	529257 (Mv: 1,000,000)	Sigma-Aldrich	Water	0.1 mg/mL	40 uL

Measurements/Analysis

AFM-IR: AFM-IR measurements were performed in the resonance-enhanced AFM-IR mode, or homodyne AFM-IR mode, with a commercial system (Dimension IconIR, Bruker). The measurements were performed with a gold-coated cantilever (PR-UM-CnIR-B, Bruker) at 25 °C with humidity below RH1%. The output of a quantum cascade laser (QCL) (Daylight, MIRCat) was pulsed at the frequency f_m, identical to the frequencies of the 7th resonances of the cantilever. The laser polarization was set to p-polarization (= parallel to the long axis of the probe). The details of the point measurement/imaging conditions are as follows: Pixel resolution = 1 cm^{-1}, Accumulation time = 60 s, Average = 5-10 times, Wavenumber range = 1800–800 cm^{-1}, scan size = 10 μm, pixel number = 128 x 128 or 192 x 192 points, scan rate = 0.4-0.5 Hz.

CLSM: Characterization of the graphene TEM grid was performed using a confocal laser scanning microscope (CLSM; VK-X3000, Keyence).

FTIR: ATR-FTIR spectra of pHEMA were obtained using a commercial FTIR spectrometer (FT/IR-4100, JASCO). Spectra were recorded in the range of 4000–650 cm^{-1} at 4 cm^{-1} resolution and averaged over 64 scans.

Software: IgorPro 9.05 was used in addition to the software equipped on the instruments.

Supplementary figures

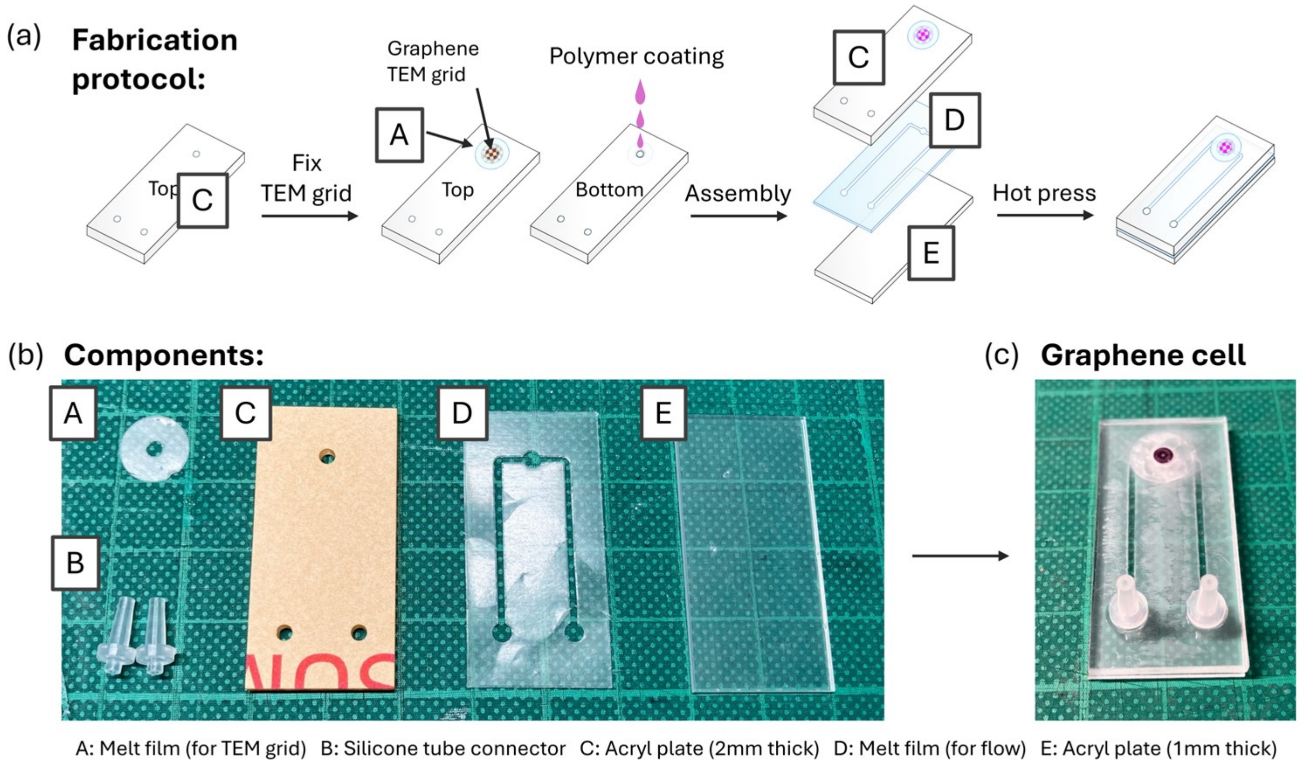

Figure S1. Fabrication protocol for microfluidic cells based on graphene TEM grids (graphene cells).

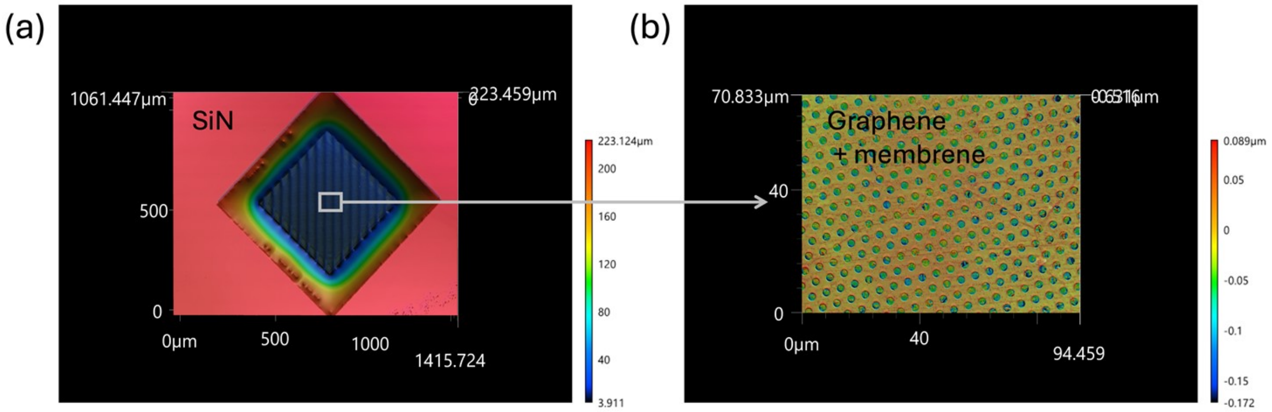

Figure S2. Laser microscope images of the graphene TEM grid used in this study.

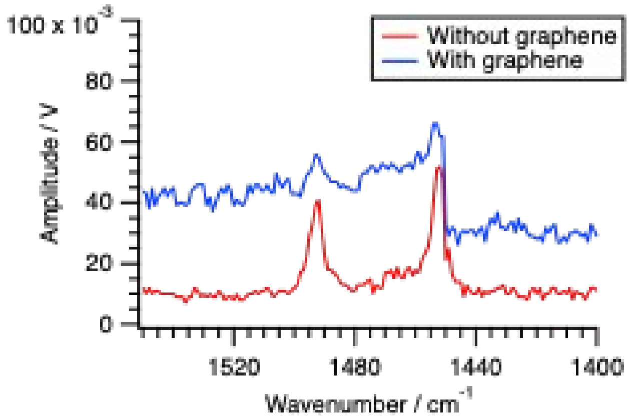

Figure S3. PTIR spectra of polystyrene (PS) beads obtained via graphene (blue) and by directly approaching the PS beads (red).

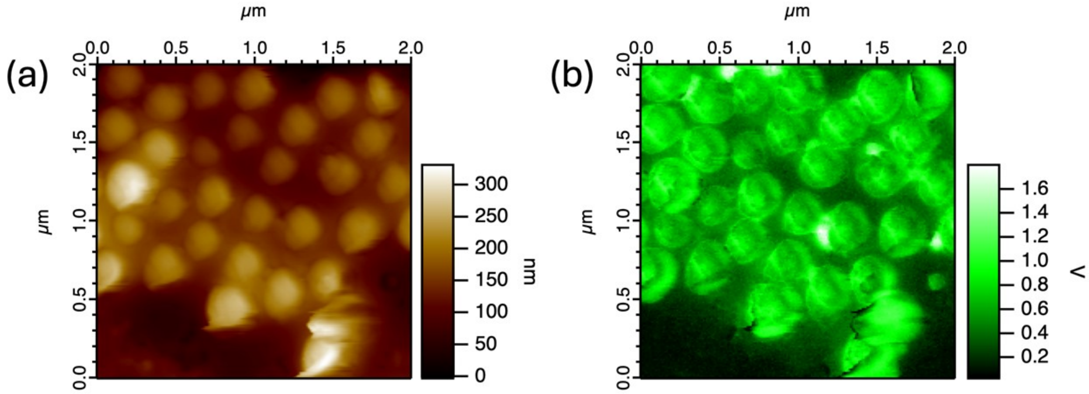

Figure S4. PTIR imaging of PS beads performed by directly approaching the PS beads. (a) AFM height of PS beads. (b) corresponding IR image at 1492 cm^{-1}.

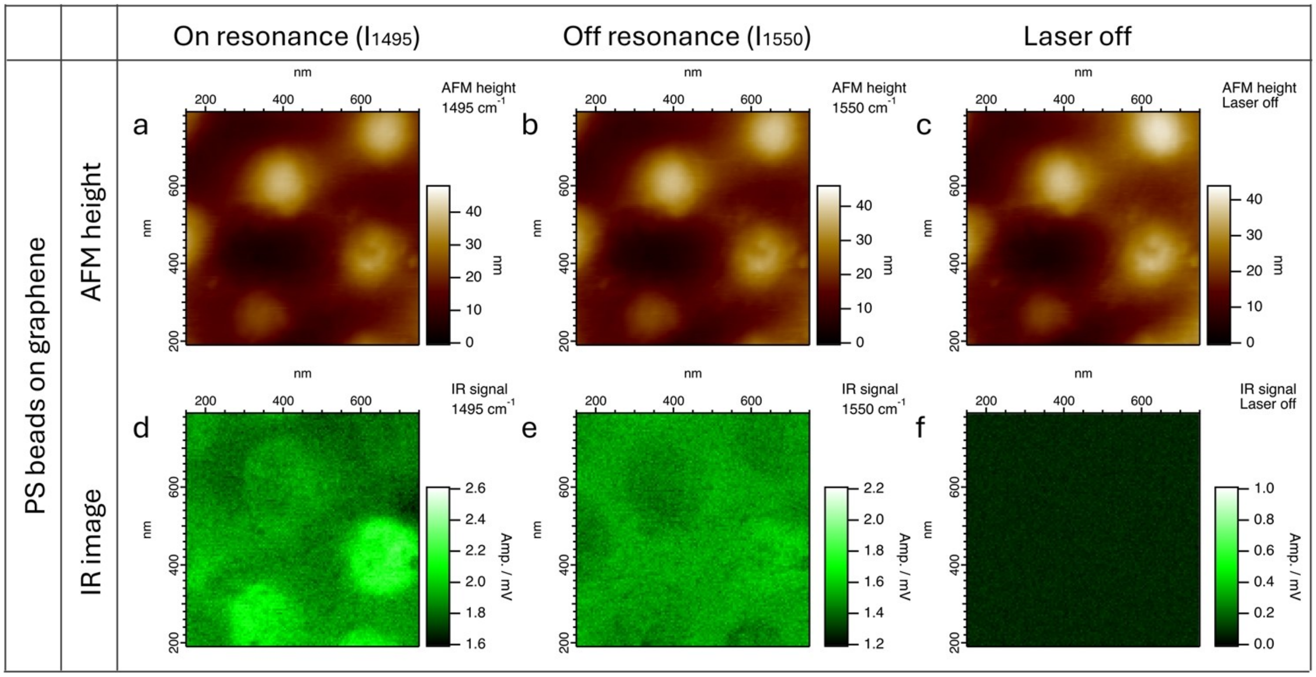

Figure S5. PTIR and corresponding AFM height images at 1495 cm^{-1} (on resonance: C=C stretching of PS + graphene broad absorption), 1550 cm^{-1} (off resonance: graphene broad absorption), and without laser irradiation.

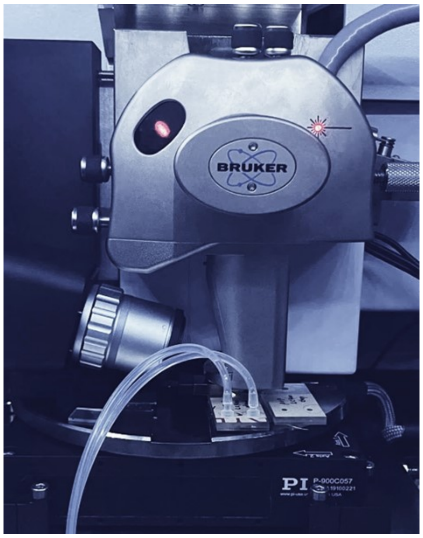

Figure S6. Photograph taken during in-situ PTIR measurements.

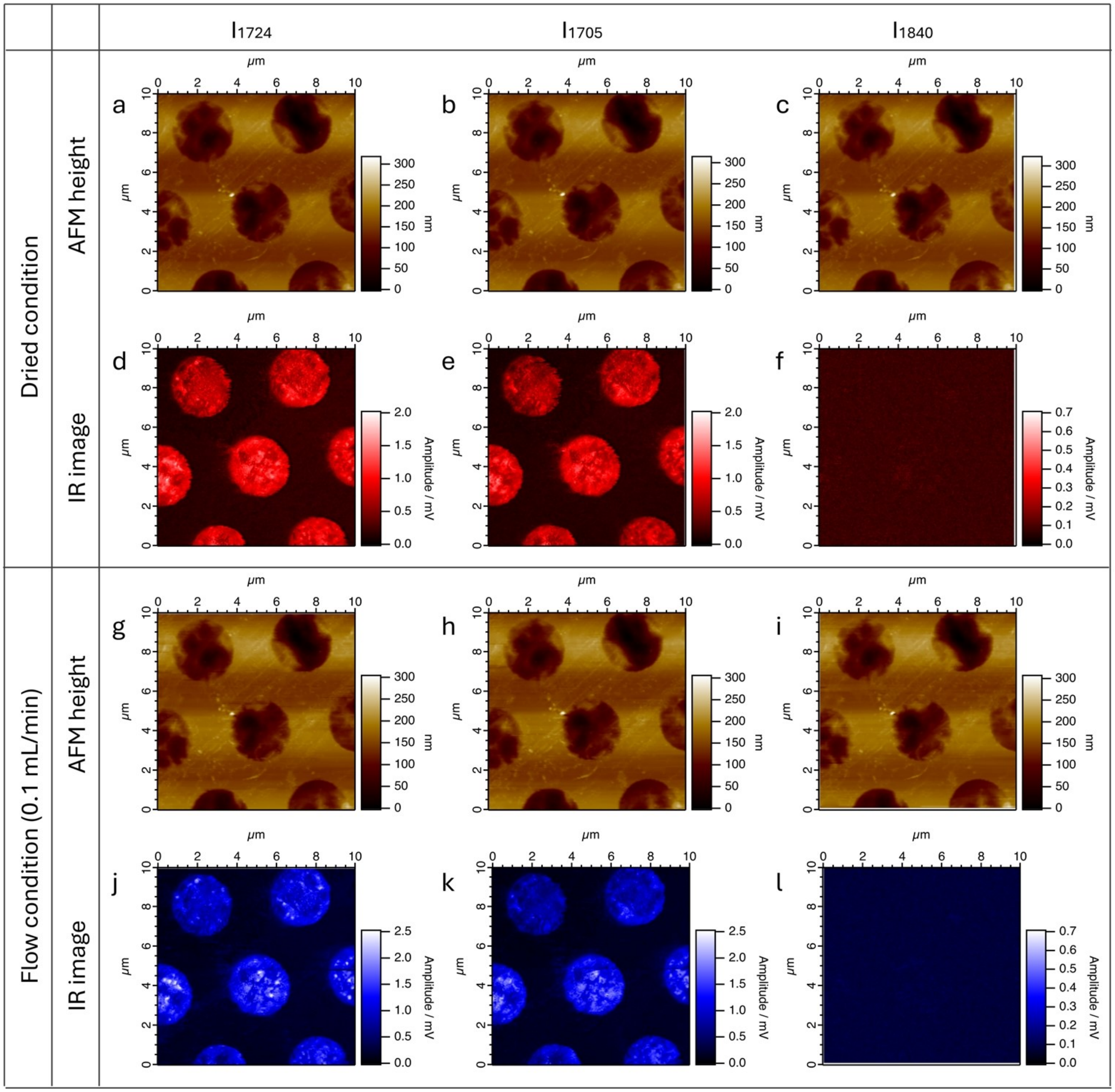

Figure S7. PTIR imaging and corresponding AFM height images at 1724 cm^{-1} (on resonance: C=O stretching of pHEMA), 1705 cm^{-1} (on resonance: hydrogen-bonded C=O stretching), 1840 cm^{-1} (off resonance) under dry (a-f) and flow conditions (g-l). A set of PTIR imaging under flow conditions of 0.1 mL/min was carried out 45 minutes after the flow began. Afterward, the sample was placed in a dry chamber (RH <1 %, 25 °C) for more than 12 hours following the removal of water from the microfluidic cell. The PTIR imaging was then conducted again under these dry conditions. All images were obtained with identical conditions for both dry and flow conditions at 0.5 Hz with a pixel resolution of 192x192 pixels.

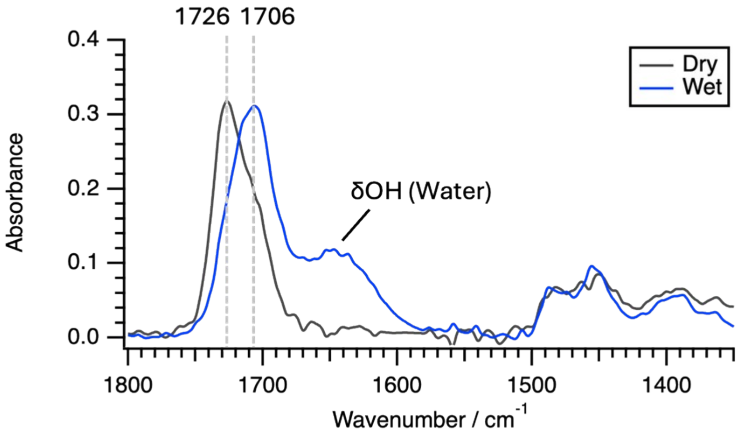

Figure S8. ATR-FTIR spectrum of pHEMA under dry and wet conditions.
